# Dynamic Protein Acetylation in Plant–Pathogen Interactions

**DOI:** 10.3389/fpls.2016.00421

**Published:** 2016-03-30

**Authors:** Gaoyuan Song, Justin W. Walley

**Affiliations:** Department of Plant Pathology and Microbiology, Iowa State University, AmesIA, USA

**Keywords:** acetylation, plant–pathogen interaction, defense, proteomics, post-translational modification

## Abstract

Pathogen infection triggers complex molecular perturbations within host cells that results in either resistance or susceptibility. Protein acetylation is an emerging biochemical modification that appears to play central roles during host–pathogen interactions. To date, research in this area has focused on two main themes linking protein acetylation to plant immune signaling. Firstly, it has been established that proper gene expression during defense responses requires modulation of histone acetylation within target gene promoter regions. Second, some pathogens can deliver effector molecules that encode acetyltransferases directly within the host cell to modify acetylation of specific host proteins. Collectively these findings suggest that the acetylation level for a range of host proteins may be modulated to alter the outcome of pathogen infection. This review will focus on summarizing our current understanding of the roles of protein acetylation in plant defense and highlight the utility of proteomics approaches to uncover the complete repertoire of acetylation changes triggered by pathogen infection.

## Introduction

Protein lysine acetylation is a reversible covalent modification that was first discovered on histones more than 50 years ago (Phillips, 1963; Allfrey et al., 1964; Verdin and Ott, 2015). In general hyperacetylation of histone proteins is associated with an open chromatin state and active transcription whereas histone deacetylation is associated with closed chromatin and a repressed transcriptional state. Additionally, histone acetylation recruits “reader” proteins (e.g., bromodomain containing proteins) that bind acetylated lysines enabling further modulation of the transcriptional state (Kouzarides, 2007; Verdin and Ott, 2015).

While protein acetylation was originally discovered on histones specifically it has long been known that non-histone proteins are also acetylated (Sterner et al., 1978; Verdin and Ott, 2015). Initially, studies focused on the role of non-histone acetylation of individual proteins. These studies demonstrated that many different types of proteins are acetylated, such as transcription factors, nuclear receptors, cytoskeletal proteins, and enzymes involved in metabolism. Additionally, acetylation was shown to modify protein function by affecting the three-dimensional structure, activity, stability, transportation and/or degradation of non-histone proteins (Glozak et al., 2005; Singh et al., 2010; Verdin and Ott, 2015). Due to the growing recognition of the importance and potential biological impact of protein acetylation a number of labs worked to develop proteomic methodology to globally detect and quantify lysine acetylation.

## Global MS Proteomics

**FIGURE 1:**
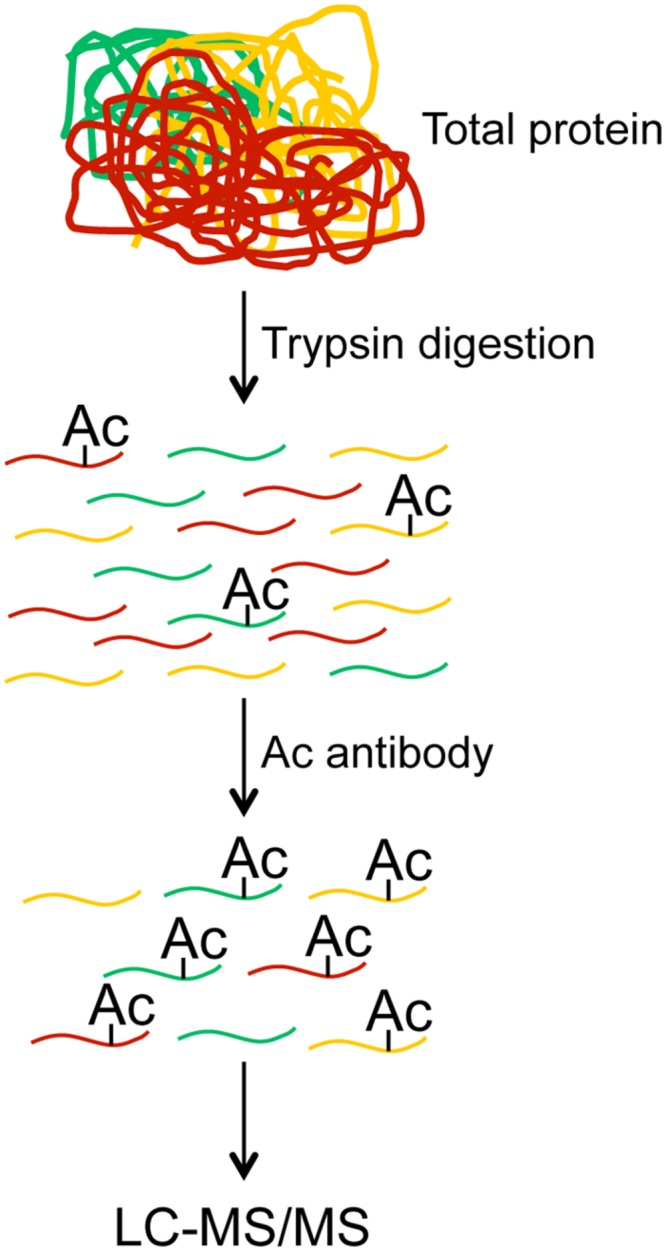
**Schematic of typical proteomic workflows for acetylome profiling**.

In the past two decades the field of MS based proteomics has rapidly matured to the point where we can routinely detect and quantify 5–10 1000 proteins in a single run (Nagaraj et al., 2011; Walley et al., 2013, 2015). However, the low abundance of acetylated proteins prevented global identification and quantification of lysine acetylation. This challenge was recently overcome by the development of pan anti-acetyllysine antibodies that recognize acetylated lysine irrespective of surrounding amino acids. By coupling acetyllysine immunopurification with MS based proteomics researchers are now able to globally profile lysine acetylation (Choudhary et al., 2009; Mertins et al., 2013). In this approach the anti-acetyllysine antibodies are employed in a technical strategy similar to the one outlined in **Figure 1**. Specifically, total proteins are extracted from cells or tissues, then the total proteins are digested to peptides, and the acetylated peptides are enriched using the anti-acetyllysine antibodies. Following enrichment the acetylated peptides are detected and quantified by using LC–MS/MS methods. Using this methodology, 100s and 1000s of acetylated sites have been identified in plants and non-plant eukaryotic systems, respectively. Organisms that have been utilized for either global or organellar acetylome profiling include *Arabidopsis* (Finkemeier et al., 2011; Wu et al., 2011; Konig et al., 2014), rice (Nallamilli et al., 2014), soybean (Smith-Hammond et al., 2014b), pea (Smith-Hammond et al., 2014a), grape (Melo-Braga et al., 2012), strawberry (Fang et al., 2015), human (Choudhary et al., 2009; Zhao et al., 2010; Barjaktarovic et al., 2015; Liu et al., 2015; Scholz et al., 2015; Wu et al., 2015), mouse (Yang et al., 2011; Chen et al., 2012; Fritz et al., 2012; Hebert et al., 2013; Masri et al., 2013; Holper et al., 2015; Kim et al., 2015), rat (Bouchut et al., 2015), *Drosophila* (Weinert et al., 2011; Feller et al., 2015), silkworm (Nie et al., 2015), yeast (Downey et al., 2015), *Toxoplasma gondii* (Xue et al., 2013), *Escherichia coli* (Zhang et al., 2013; Castano-Cerezo et al., 2014), and other bacteria (Okanishi et al., 2013; Wu et al., 2013; Liao et al., 2014; Liu et al., 2014; Pan et al., 2014; Kosono et al., 2015; Mo et al., 2015; Xie et al., 2015). Collectively, these studies demonstrate that non-histone acetylation is a common modification in different systems and suggest that acetylation plays and essential role in a myriad of biological processes.

## Enzymatic and Non-Enzymatic Ac

Lysine acetylation is typically regulated by enzymes that add or remove acetyl groups. Specifically, lysine acetyltransferases (also termed HATs) have been shown to acetylate both histone and non-histone proteins (Sterner and Berger, 2000). Lysine acetyltransferases are divided, based on homology, into three different families, GNAT, MYST, and CBP/P300 (Kouzarides, 2007). Conversely, acetyl groups are removed from the acetylated proteins by lysine deacetylases (also termed HDACs; Kouzarides, 2007; Haery et al., 2015). Thus, protein acetylation levels are dynamically regulated by lysine acetyltransferases and deacetylases. Intriguingly, recent studies have demonstrated that protein acetylation is not only controlled enzymatically, but that it is also modulated non-enzymatically by metabolic intermediates including Acetyl-CoA and NAD^+^, which is required for activity of sirtuin type deacetylases (Choudhary et al., 2009; Cai et al., 2011; Lu and Thompson, 2012; Shen et al., 2015).

## Histone Ac and Defense in Plants

**FIGURE 2:**
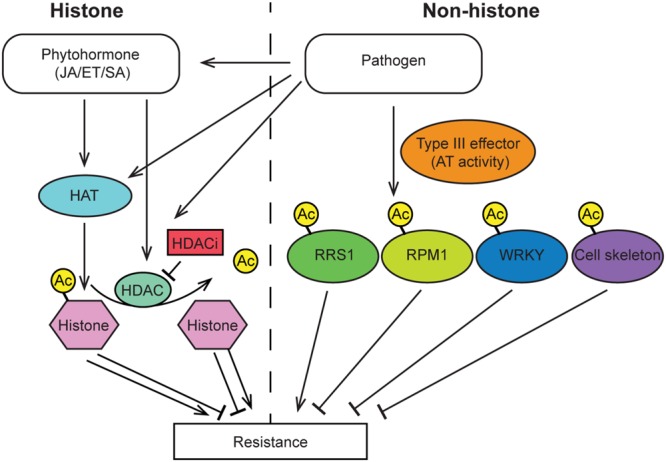
**Overview of histone and non-histone protein acetylation events that have been demonstrated to alter plant immunity.** Pathogen infection results in modulation of HAT and HDAC activity, which alters the histone acetylation state of specific defense gene promoters thereby promoting either susceptibility or resistance. Several pathogen effector proteins encode acetyltransferase enzymes that directly acetylate host proteins and alter plant immunity. JA, jasmonic acid; ET, ethylene; SA, salicylic acid; HAT, histone acetyltransferase; HDAC, histone deacetylase; HDACi, histone deacetylase inhibitor; RRS1, Toll/Interleukin1 receptor type R protein; RPM1, intracellular nucleotide binding-leucine-rich repeat R protein.

Acetylation is a common modification of histones 3 and 4. Generally, histone acetylation is enriched in the promoter region of genes, which functions to open the chromatin and enable gene expression (**Figure 2**). Studies have found that the expression level of HAT genes is induced by treatment with hormones as well as pathogen infection (Liu et al., 2012; Xu et al., 2015). Consistently, the level and pattern of histone acetylation is altered by pathogen infection. Finally, the maize fungal pathogen *Cochliobolus carbonum* produces the effector molecule HC-toxin, which functions as a HDACi and is required for pathogen virulence (Johal and Briggs, 1992; Brosch et al., 1995; Ransom and Walton, 1997; Sindhu et al., 2008). Taken together these studies indicate that histone acetylation levels may play an important role in defense.

In line with these observations, a direct role for HDACs in modulating histone acetylation of defense genes and thereby plant resistance has been shown. The transcription level of Histone Deacetylase701 (*HDT701*), a member of the plant-specific HD2 subfamily of HDACs in rice, is increased in the compatible reaction and decreased in the incompatible reaction after infection by the fungal pathogen *Magnaporthe oryzae* (Ding et al., 2012). Critically, overexpression of *HDT701* in transgenic rice leads to decreased levels of histone H4 acetylation and enhanced susceptibility to the rice pathogens *M. oryzae* and *Xanthomonas oryzae* pv. *oryzae* (*Xoo*). In contrast, silencing of *HDT701* in transgenic rice causes elevated levels of histone H4 acetylation and elevated transcription of PRR and defense-related genes, increased generation of reactive oxygen species after pathogen-associated molecular pattern elicitor treatment, as well as enhanced resistance to both *M. oryzae* and *Xoo*.

In *Arabidopsis*, the RPD3-type histone deacetylases HDA6 and HDA19 have been extensively studied in the context of plant immunity. HDA19 modulates histone acetylation and is a positive regulator of JA/ET signaling pathways. Consistently, HDA19 modulates resistance to pathogens sensitive to JA/ET mediated immunity such as *Alternaria brassicicola* (Zhou et al., 2005). Conversely, HDA19 appears to play a negative role in SA mediated signaling and defense (Choi et al., 2012). Furthermore, HDA19 interacts with the transcription factors WRKY38 and WRKY62, which are negative regulators of SA defense signaling, to fine tune basal defense responses (Kim et al., 2008). Finally, HDA6 acts as a corepressor with JA-Zim domain (JAZ) proteins to repress EIN3/EIL1 dependent transcriptional responses and thereby JA signaling.

Histone acetyltransfersases have also been demonstrated to modulate *Arabidopsis* immunity via Elongator mediated gene regulation. Recent studies established that *Arabidopsis* Elongator complex mutants exhibit altered histone acetylation and target gene expression patterns, leading to altered pathogen resistance phenotypes. Specifically, mutation of Elongator subunit 2 (ELP2) reduces the histone acetylation level in the coding region of several plant defense genes, including *PLANT DEFENSIN1.2 (PDF1.2), WRKY33* and *OCTADECANOID-RESPONSIVE ARABIDOPSIS AP2/ERF59 (ORA59)*, which results in the repression of these genes and leads to suppression of plant defense (Wang et al., 2013, 2015). Mutation of another Elongator complex subunit, AtELP3, results in similar immune deficiencies as ELP2. AtELP3 has also been demonstrated to have HAT activity. Collectively these findings indicate that the Elongator complex is involved in both basal immunity and ETI (Defraia et al., 2013).

## Non-Histone Ac in Plant Immunity

While the majority of research has investigated the role of histone acetylation in defense there are several studies that have established a role for non-histone protein acetylation in plant immunity. For instance, pathogen produced type III effectors can acetylate selected non-histone proteins in host cells, thereby triggering plant immunity response (**Figure 2**). One example of such an interaction is from the type III secreted effector, HopZ1a, which is secreted by *Pseudomonas syringae* and functions as an acetyltransferase. HopZ1 is able to self-acetylate and acetylate tubulin, which results in the destruction of plant microtubule networks, inhibits protein secretion and ultimately suppresses cell-wall mediated defense (Lee et al., 2012). A similar mechanism was found for the effector protein AvrBsT that also acts as an acetyltransferase and acetylates ACIP1, which is required for plant defense. Acetylation of ACIP1 alters the co-localization pattern of ACIP1 and microtubules (Cheong et al., 2014). Additionally, another type III secreted effector, PopP2, a YopJ-like effector from the soil borne root pathogen *Ralstonia solanacearum*, also has acetyltransferase activity. PopP2 directly acetylates the C-terminal WRKY transcription factor domain of the NLR RRS1 as well as WRKY transcription factors. Acetylation of RRS1 or WRKYs abolishes DNA binding activity. In the case of RRS1 acetylation, RPS4 dependent immunity is activated. Conversely, acetylation of WRKY transcription factors results in immune suppression (Tasset et al., 2010; Le Roux et al., 2015; Sarris et al., 2015). Finally, a new type III effecter HopZ3 from the plant pathogen *P. syringae*, have been found to acetylate multiple members of the RPM1 immune complex and depress plant immunity response (Lee et al., 2015).

## Perspective

Lysine acetylation has emerged as a major post-translational modification impacting a diverse array of cellular processes. In the context of plant defense signaling it is well established that modulation of histone acetylation levels of defense genes is critical for appropriate immune responses. There is also mounting evidence that acetylation of non-histone proteins impacts plant immunity. However, to date non-histone protein acetylation of plant proteins has only been shown to result from pathogen effectors that encode acetyltransferase enzymes. This raises the question of whether plant HATs or HDACs directly modulate acetylation status of non-histone proteins during pathogen infection. The recent development of robust proteomic methodologies to globally identify and quantify lysine acetylation enables us to now identify proteome-wide changes in acetylation triggered during immune responses. Detailed follow-up studies of specific alterations in acetylation triggered by pathogen infection should shed light on whether plants directly modulate non-histone protein acetylation during defense signaling.

## Author Contributions

All authors listed, have made substantial, direct and intellectual contribution to the work, and approved it for publication.

## Conflict of Interest Statement

The authors declare that the research was conducted in the absence of any commercial or financial relationships that could be construed as a potential conflict of interest.

## Abbreviations

ET: Ethylene
ETI: effector-triggered immunity
HATs: histone acetyltransfersases
HDACs: histone deactylases
HDACi: histone deacetylase inhibitor
JA: jasmonic acid
LC–MS/MS: liquid chromatography tandem mass spectrometry
MS: mass spectrometry
NLR: nucleotide-binding/leucine-rich repeat receptor
PRR: pattern recognition receptor
SA: salicylic acid
